# Musculoskeletal Pitfalls on Molecular Imaging Studies of Oncologic Patients: How to Stay Out of Trouble

**DOI:** 10.3390/tomography10030030

**Published:** 2024-03-08

**Authors:** Brooke Sarna, Ty Subhawong, Efrosyni Sfakianaki, Richard Wang, Anna Christodoulou-Vega, Fabiano N. Cardoso

**Affiliations:** 1 Department of Radiology, University of Miami Miller School of Medicine, Miami, FL 33136, USA; 2 Department of Nuclear Medicine, University of Miami Miller School of Medicine, Miami, FL 33136, USA

**Keywords:** benign tumors, CT scan, incidental findings, molecular imaging, MR imaging, musculoskeletal, nuclear medicine, oncology, PET scan, pitfalls, radiography

## Abstract

An increasing amount of molecular imaging studies are ordered each year for an oncologic population that continues to expand and increase in age. The importance of these studies in dictating further care for oncologic patients underscores the necessity of differentiating benign from malignant findings, particularly for a population in whom incidental findings are common. The aim of this review is to provide pictorial examples of benign musculoskeletal pathologies which may be found on molecular imaging and which may be mistaken for malignant processes. Imaging examples are provided in the form of radiographs, bone scintigraphy, computed tomography, and fluorine-18 fluorodeoxyglucose positron emission tomography/computed tomography (FDG PET/CT) scans. Special attention is paid to specific features that help narrow the differential diagnosis and distinguish benign from malignant processes, with the goal of avoiding unnecessary invasive procedures.

## 1. Introduction

Nuclear medicine imaging, in particular bone scintigraphy and positron emission tomography (PET), plays a critical role in the diagnosis and management of oncology patients—a role that will only continue to grow in the future. In the next ten years, the number of individuals living at least ten years after their cancer diagnosis is projected to increase by 22.4%, and 74% of cancer survivors will be over the age of 65 [1].

Alongside treatment advancements, imaging techniques continue to evolve as well. Computed tomography (CT) and magnetic resonance imaging (MRI) can now be combined with PET for high-resolution full-body scanning. The emergence of targeted radiotracers has enhanced detection of particular cancers, and improved scanning hardware and image reconstruction algorithms have reduced exam times with less radiation and fewer artifacts [2].

With the continued growth of molecular imaging of oncology patients, the dilemma of incidental findings becomes of increasing concern. In this paper, we aim to provide a pictorial review of both common and uncommon benign musculoskeletal pathologies that may be incidentally found on bone scintigraphy and PET scans, and which may mimic malignancies. In many of the illustrated cases, biopsies were performed that may have otherwise been avoided with increased recognition of these benign pathologies. Familiarity with their key imaging features would improve staging accuracy and guide appropriate management. All of the images included in this work are sourced from the authors’ own databases.

## 2. Bone

### 2.1. Fracture

Incidental insufficiency fractures (IFs) are increasingly detected on surveillance and re-staging studies as therapeutic advances continue to extend the lives of oncology patients [3]. Factors including osteoporosis, malnutrition, and frailty are associated with higher risk of acute fractures [3], with chemotherapy and radiotherapy (RT) in particular predisposing oncology patients to IFs. Although the exact incidence after RT is not known, prior studies reveal a wide range of incidences from 8 to 45% [4], and there is correlation with age and type of cancer treated [4,5]. In some studies, evidence of IFs were found in up to 89% of post-RT MRIs and 34% of post-RT bone scans [6,7].

**Figure 1:**
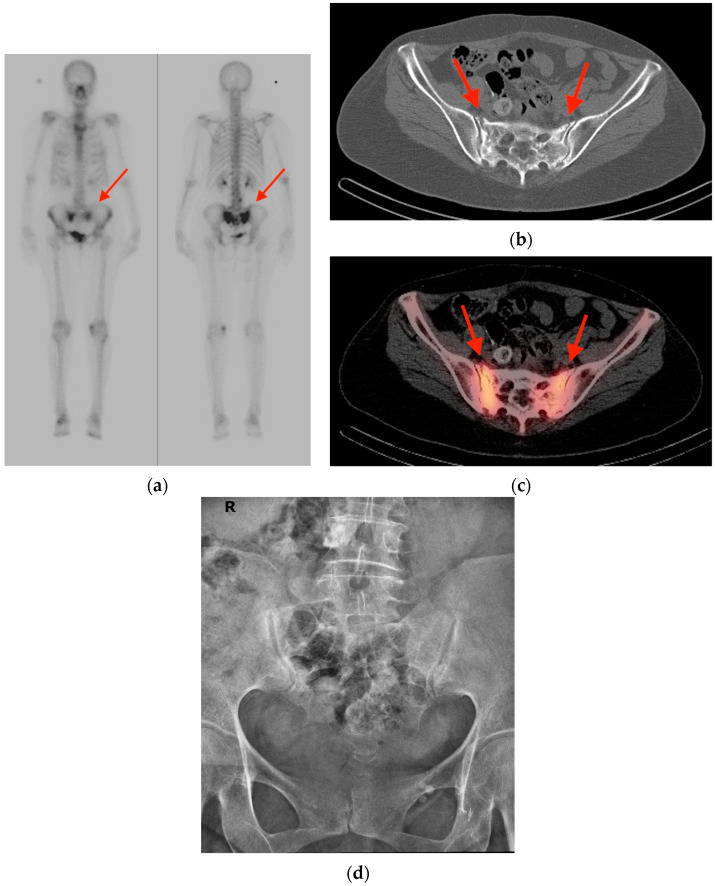
(**a**) Nuclear medicine bone scintigraphy scan obtained in a 62-year-old woman with colon adenocarcinoma and complaints of lower back pain shows a classic “H-sign” of focal uptake in the region of the sacrum (red arrows). (**b**) CT scan of the same patient shows cortical irregularities along the anterior aspect of the sacrum bilaterally (red arrows), consistent with fracture. (**c**) Fused PET/CT imaging reveals focal radiotracer uptake in the region of the fractures (red arrows). (**d**) Radiograph obtained in the course of work-up revealed no obvious abnormalities (R = right side of patient). CT = computed tomography. PET/CT = positron emission tomography/computed tomography.

Fractures may persist for a minimum of 5 months on nuclear medicine bone scans, with only 90% fully resolving after 2 years [8]. The Honda sign (or “H” sign) has been described as a classic presentation of sacral IFs on bone scans, although it may only be found in less than half of all cases [9] (Figure 1). Characteristics of IFs on combined positron emission tomography/computed tomography (PET/CT) vary with age and location; however, the mean standard uptake value (SUV) tends to range between 1.4 and 2.5 [10,11]. In contrast, Shin et al. showed a maximum mean SUV (SUVmax) of 12.0 among 19 malignant pathologic fractures [12]. In equivocal cases of sacral IFs, MRI can be helpful in delineating distinguishing features such as vertically oriented trabecular fracture lines on fat-suppressed coronal T2-weighted sequences [13].

### 2.2. Fibrous Dysplasia

**Figure 2:**
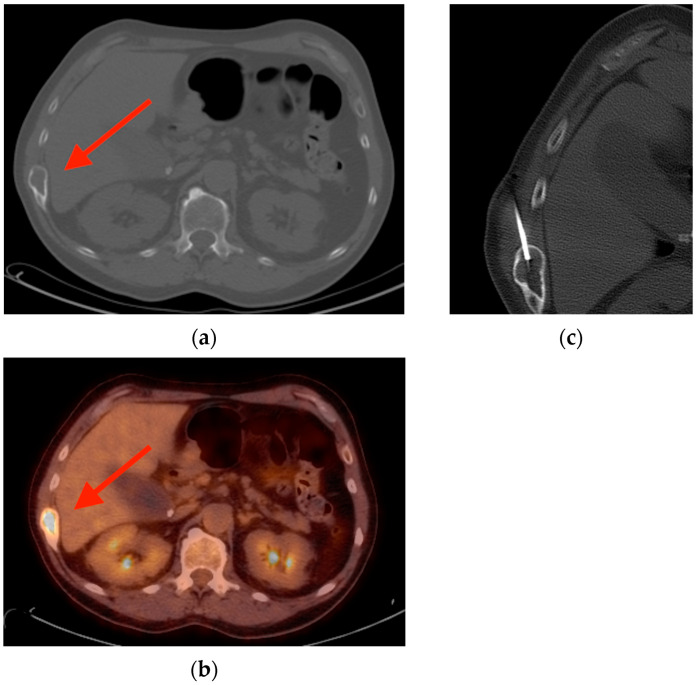
(**a**) CT scan of a 61-year-old patient with head and neck squamous cell carcinoma reveals an expansile lesion in the right rib (red arrow) with non-aggressive features including a well-defined sclerotic border and no periosteal reaction. (**b**) Corresponding FDG PET/CT imaging shows focal uptake in this same lesion (red arrow). (**c**) Despite its features, this lesion was later biopsied and found to be fibrous dysplasia. CT = computed tomography. FDG PET/CT = fluorine-18 fluorodeoxyglucose positron emission tomography/computed tomography.

**Figure 3:**
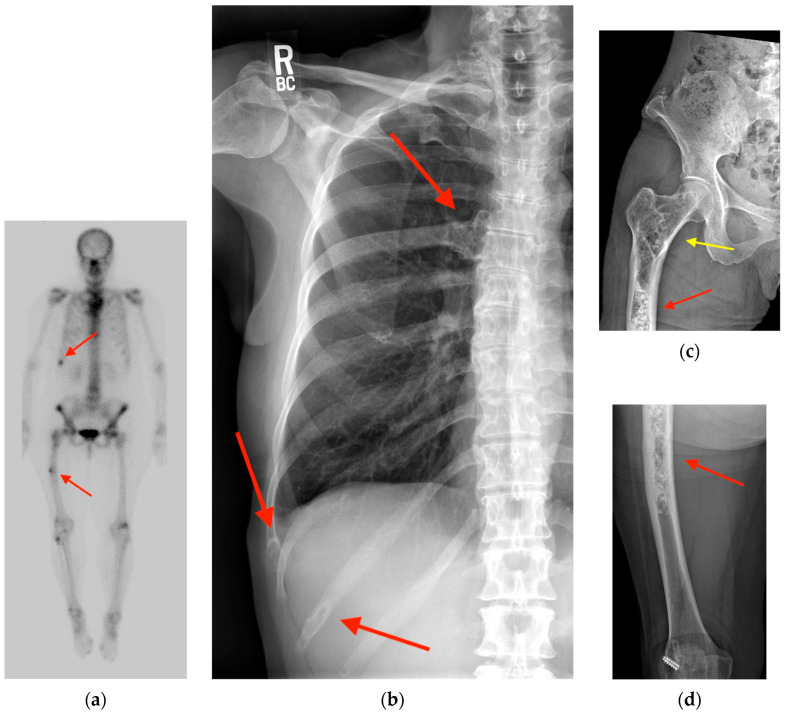
(**a**) Bone scintigraphy in a 69-year-old woman with urothelial carcinoma shows multiple areas of focal uptake including in the right eleventh rib and the right femur (red arrows). (**b**) Corresponding chest radiograph reveals multiple “ground glass” expansile lesions in the ribs (red arrows) surrounded by a halo of sclerosis suggestive of polyostotic fibrous dysplasia. Radiographs of the proximal right femur (**c**) and distal right femur (**d**) reveal a long intramedullary lesion (red arrows) with a classic Shepherd crook deformity (yellow arrow). This lesion has a denser matrix, correlating with a higher degree of woven bone relative to fibrous elements.

Fibrous dysplasia (FD) is a benign mesenchymal tumor caused by a post-zygotic missense mutation in the *GNAS* gene resulting in the proliferation of fibroblast-like spindle cells associated with immature woven bone [14]. About 80% of lesions occur in only one bone (monostotic) (Figure 2) with a minority of cases occurring in multiple bones (polyostotic) (Figure 3). The most common locations include the ribs, followed by the femurs, and finally the craniofacial bones [15]. FD lesions display focal uptake on bone scintigraphy but can have variable uptake on fluorine-18 fluorodeoxyglucose PET/CT (in this paper abbreviated as FDG PET/CT), ranging from 1.2 to 9.6 [16], which likely depends on the amount of active fibroblasts. The lesions may also show uptake on prostate-specific membrane antigen (PSMA) PET/CT imaging [17]. While a thick sclerotic rim and “ground-glass” matrix of woven bone are characteristic of FD, imaging manifestations are protean and may represent only one component of a complex multi-organ disease [18,19].

### 2.3. Enchondroma

**Figure 4:**
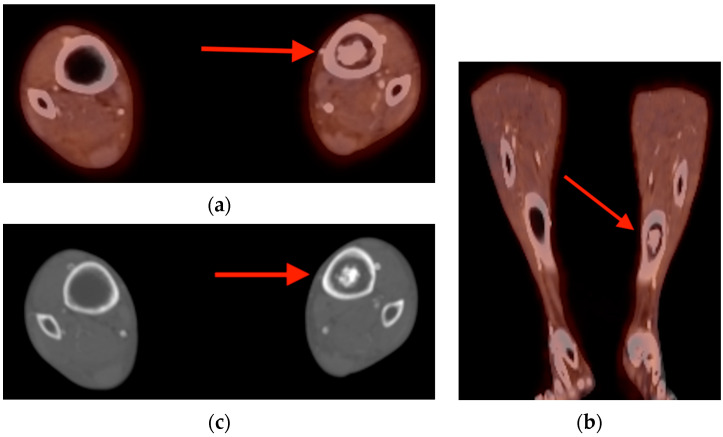
Axial (**a**) and coronal (**b**) fused FDG PET/CT images obtained in a 62-year-old man with melanoma showed a coarsely calcified intramedullary bone lesion with mild uptake within the left tibia (red arrows). (**c**) Plain CT images show the chondroid matrix calcifications and irregular but narrow zone of transition bordering this enchondroma in better detail (red arrow). CT = computed tomography. FDG PET/CT = fluorine-18 fluorodeoxyglucose positron emission tomography/computed tomography.

Enchondromas are the most common cartilaginous bone tumors and comprise roughly 13% of benign bone tumors [20]. The most common features include “ring and arc” or stippled patterns of chondroid calcification, with well-defined margins and little, if any, endosteal scalloping [21]. In the small tubular bones of the hands and feet, enchondromas may lack matrix mineralization [22]. Like many benign bone tumors, enchondromas may display focal uptake on bone scintigraphy and FDG PET/CT (Figure 4). Distinguishing enchondroma and chondrosarcoma (particularly when they are low-grade) remains a diagnostic dilemma [23], although enchondromas tend to have less intense radiotracer uptake on both bone scans and FDG PET with an average SUVmax of 1.6 versus 4.4 for chondrosarcoma [24]. While some features overlap, findings of deep endosteal scalloping, cortical breakthrough, bony expansion, and paucity of matrix calcifications should raise concern for atypical enchondroma/low-grade chondrosarcoma [23,25]. In general, increased uptake, periosteal reaction, cortical defects, associated soft tissue masses, and peritumoral edema should prompt concern for a malignant lesion [26], but otherwise incidentally discovered asymptomatic long bone enchondromas rarely warrant additional work-up [27].

### 2.4. Schmorl’s Node

**Figure 5:**
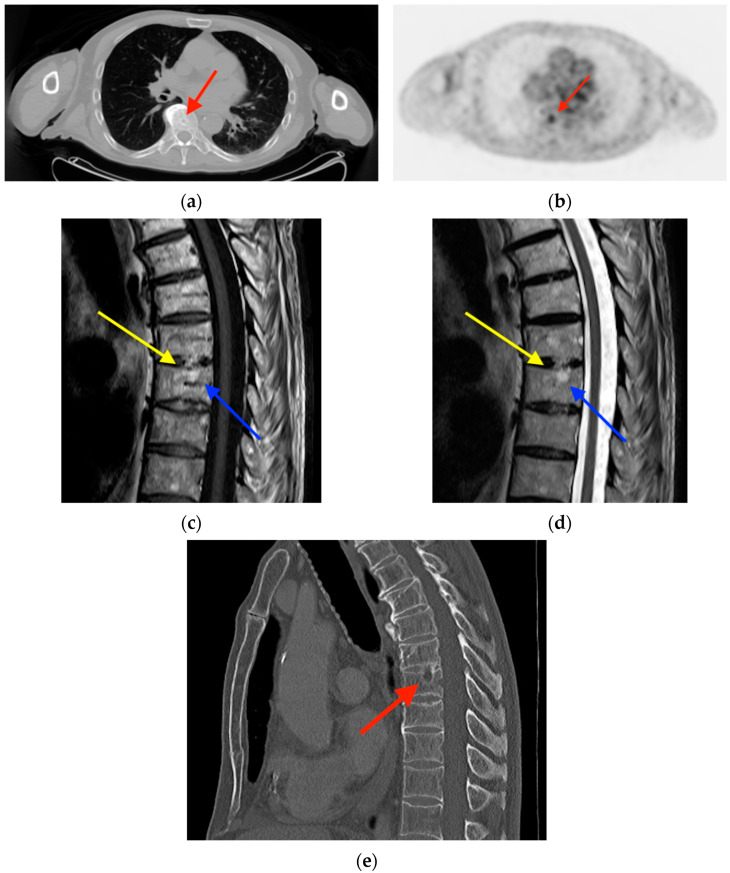
Initial CT (**a**) and FDG PET images (**b**) obtained in a 72-year-old man with head and neck squamous cell carcinoma demonstrated a lucent lesion in the T8 vertebral body corresponding with a subtle area of focal radiotracer uptake (red arrows). Sagittal T1-weighted (**c**) and T2-weighted (**d**) MRI images obtained three days after the FDG PET/CT show the bony defect at the superior end plate of T8 (yellow arrows) with intraosseous herniation of the intravertebral disk (blue arrows). (**e**) CT obtained 37 months after the initial FDG PET/CT shows a persistent lesion (red arrow), in contrast to acute calcific discitis, in which the lesion typically resolves. CT = computed tomography. FDG PET/CT = fluorine-18 fluorodeoxyglucose positron emission tomography/computed tomography.

A Schmorl’s node is an intraspongious herniation of the nucleus pulposus through the cartilaginous and bony endplate of adjacent vertebrae. With prevalence in the general population as high as 76% [28], they are often considered incidental findings, though they may be symptomatic in some cases [29]. The formation of a Schmorl’s node entails localized inflammation and osteonecrosis [30], resulting in positive focal uptake on bone scintigraphy and FDG PET/CT that can be mistaken for metastatic disease (Figure 5). As they progress through their natural history, these nodes may have SUVs ranging from 1.09 [31] to as high as 5.7 [32], and can also cause focal uptake on DOTATATE PET/CT imaging [33]. Compared to vertebral metastases, Schmorl’s nodes may show higher peripheral FDG uptake and may show a mismatch between CT and PET in which the area of uptake on FDG PET appears smaller in size than the lesion on CT [32].

### 2.5. Vertebral Hemangioma

**Figure 6:**
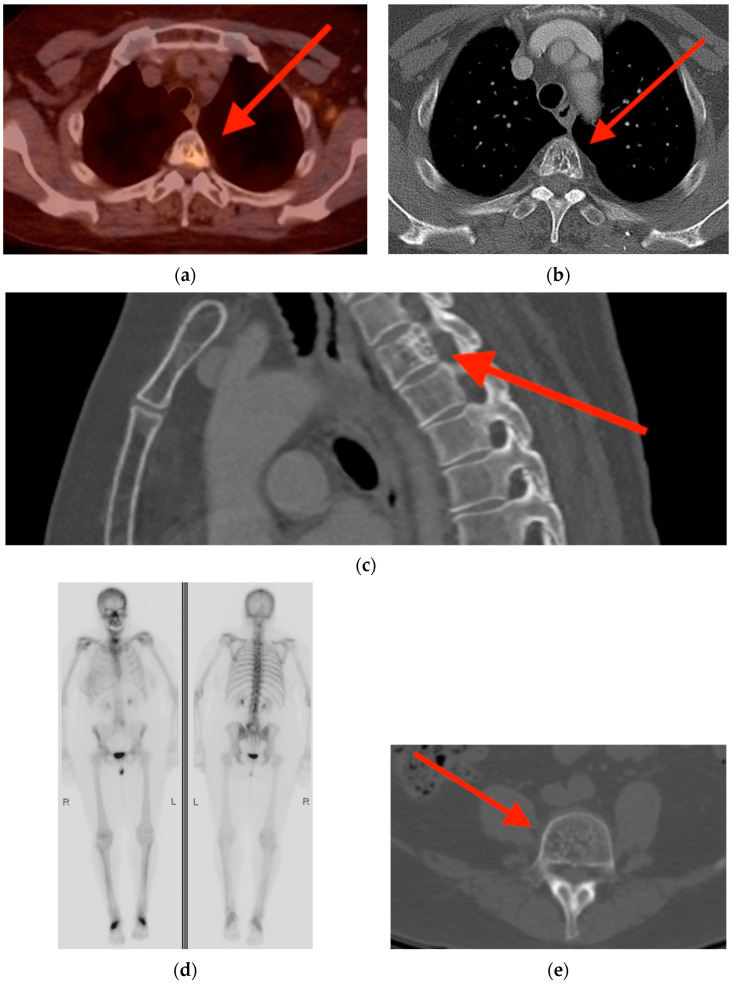
(**a**) DOTATATE PET/CT in a 74-year-old patient with small bowel neuroendocrine tumor showed focal uptake in the T4 vertebral body, with a maximum SUV of 4.1 (red arrow). Corresponding axial (**b**) and sagittal reformat (**c**) CT images show a classic “corduroy” or “polka-dot” appearance (red arrows). The Hounsfield unit (HU) measurement of this lesion averaged −119, indicating predominantly fat content. (**d**) Bone scintigraphy obtained in the course of work-up showed no vertebral abnormalities, as is usually the case for vertebral hemangiomas (VH). (**e**) CT obtained in a different patient better illustrates the “polka dot” or “corduroy” pattern that characterizes VHs (red arrow). PET/CT = positron emission tomography/computed tomography. SUV = mean standard uptake value.

Vertebral hemangiomas (VH) are the most common primary spine tumor and may occur in up to 11% of individuals [34,35]. Due to their heterogenous make-up of fat and vessels, they can have a wide range of appearances ranging from typical quiescent lesions to atypical lesions with rapid growth, interstitial edema, and extension beyond the vertebral body [36]. Lesions with rapid growth in particular may raise suspicion for atypical hemangiomas such as epithelioid hemangioendothelioma [37]. Though VHs also display varying appearance on nuclear medicine imaging, VHs are one of the few lesions discussed in this review that may appear photopenic on bone scintigraphy [38,39]. The comparatively variable nature of VHs can result in a wide array of combined bone scan and FDG PET/CT findings, including increased uptake on both, decreased uptake on both, and increased uptake on FDG PET/CT with normal uptake on bone scan [40]. Despite this variability, they can be distinguished by a “polka dot” or “corduroy” appearance on CT scan, and the preservation of intralesional fat on MRI [36] (Figure 6).

### 2.6. Paget’s Disease

**Figure 7:**
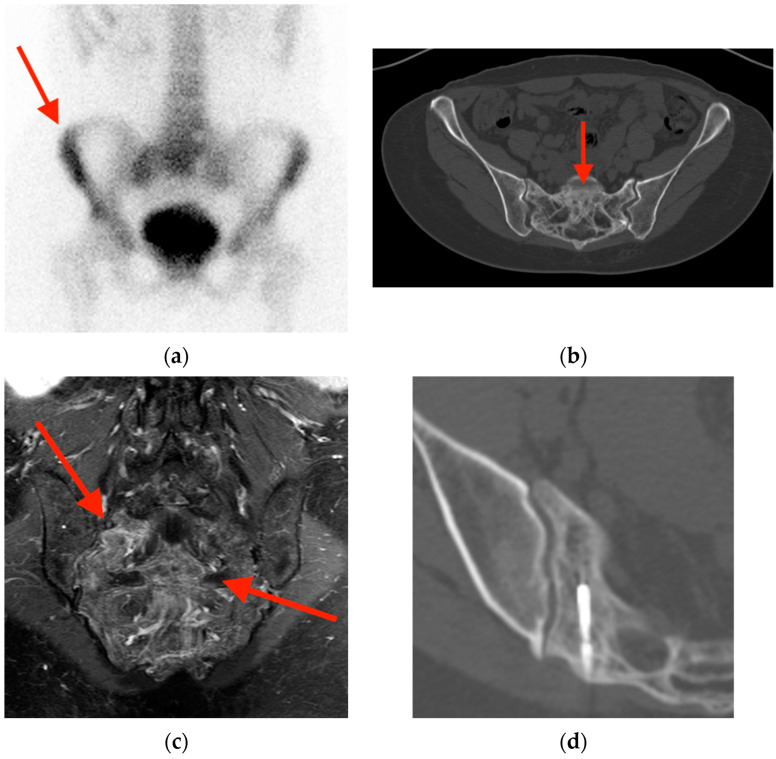
(**a**) Bone scintigraphy in a 54-year-old woman with elevated alkaline phosphatase reveals heterogeneously increased uptake in the sacrum, most prominent at the right sacral ala (red arrow). (**b**) CT shows diffuse cortical and trabecular thickening with mild bone expansion (red arrow). (**c**) Post-gadolinium fat-suppressed coronal T1-weighted MRI shows diffuse bone enhancement with associated coarse trabecular thickening (red arrows). (**d**) During the course of work-up, the sacrum was biopsied and pathology revealed active Paget’s disease. CT = computed tomography. MRI = magnetic resonance imaging.

**Figure 8:**
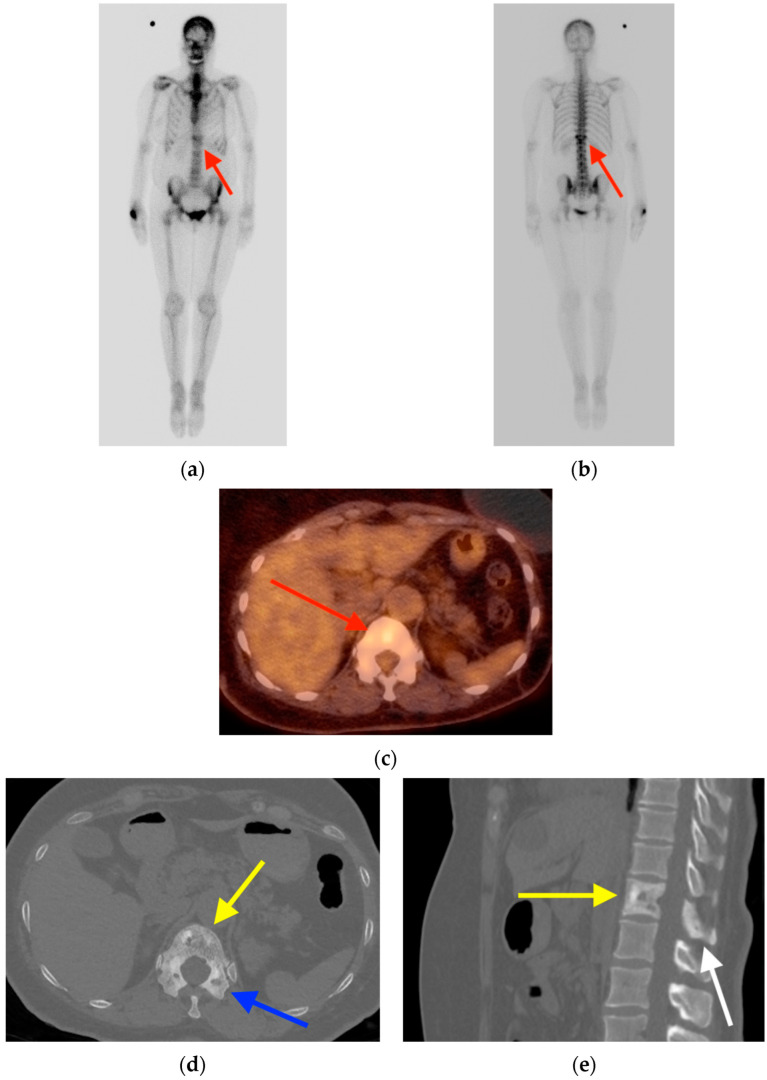
(**a**,**b**) Bone scintigraphy in a 64-year-old women with breast cancer shows focal uptake in the T12 vertebral body (red arrows). (**c**) FDG PET/CT also revealed focal radiotracer uptake (red arrow). Axial (**d**) and sagittal reformat (**e**) CT images show diffuse trabecular thickening involving the vertebral body (yellow arrows), pedicles (blue arrow), and posterior elements (white arrow), classic for Paget’s disease. CT = computed tomography. FDG PET/CT = fluorine-18 fluorodeoxyglucose positron emission tomography/computed tomography.

Paget’s disease of bone (PD) is characterized by disordered bone remodeling, commonly affecting the pelvis, lower extremities, lumbar spine, and skull, with prevalence as high as 5% in women and 8% in men by 70 years of age [41]. It rarely occurs in those under 55 years old. Unlike other incidental bone lesions, PD tends to be non-geographic and usually affects the entirety of a bone. Depending on the stage in the disease’s natural history, there is variable uptake on bone scan, and a bone scan may be positive before any radiography findings are evident [42]. Similarly, FDG and PSMA PET/CT may show diffuse low to moderate SUV uptake, with SUVmax typically > 5 [21,43,44] (Figure 7). CT reveals diffuse trabecular and cortical thickening and bony expansion. When PD occurs in isolated vertebrae, it may be easily mistaken for an osteoblastic metastatic process (Figure 8).

## 3. Muscle and Tendon

### 3.1. Enthesopathy

**Figure 9:**
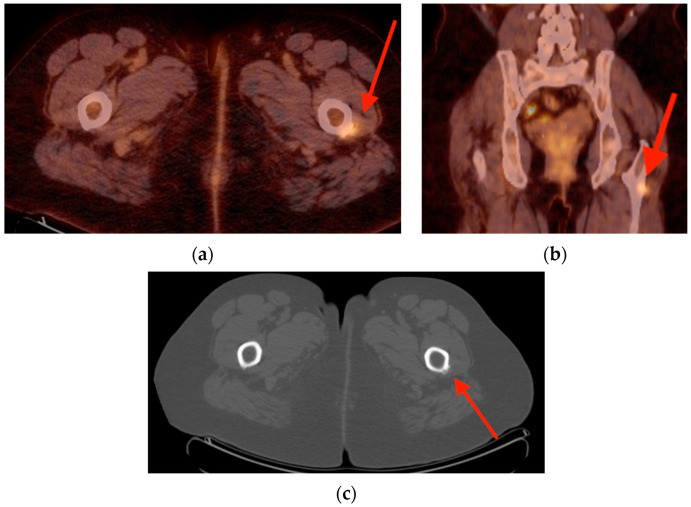
Axial (**a**) and coronal (**b**) FDG PET/CT images from a 63-year-old woman with head and neck adenocarcinoma show focal uptake in the region of the vastus lateralis origin, with SUVmax 3.7 (red arrows). (**c**) Plain CT images show periosteal changes consistent with enthesopathic changes of the vastus lateralis (red arrow). CT = computed tomography. FDG PET/CT = fluorine-18 fluorodeoxyglucose positron emission tomography/computed tomography. SUVmax = maximum standard uptake value.

Enthesopathy represents a pathologic process occurring at the sites of tendon, capsule, fascia, or ligament insertions. Its etiology can be multifactorial and includes degenerative changes, trauma, or inflammation (for example, related to rheumatoid arthritis or ankylosing spondylitis) [45]. As an inflammatory process involving hyperostosis, sites of enthesopathy will be apparent as areas of focal radiotracer uptake in areas of ligamentous and tendinous attachments on bone scintigraphy and FDG PET/CT (Figure 9). In fact, these areas may display uptake on FDG PET/CT before any other radiologically apparent findings, with SUVs typically in the range of 1.02–1.35 in early stages [46]. In patients with concomitant rheumatoid arthritis or ankylosing spondylitis, these values may be even higher, up to 4.76 [46].

### 3.2. Myositis Ossificans

**Figure 10:**
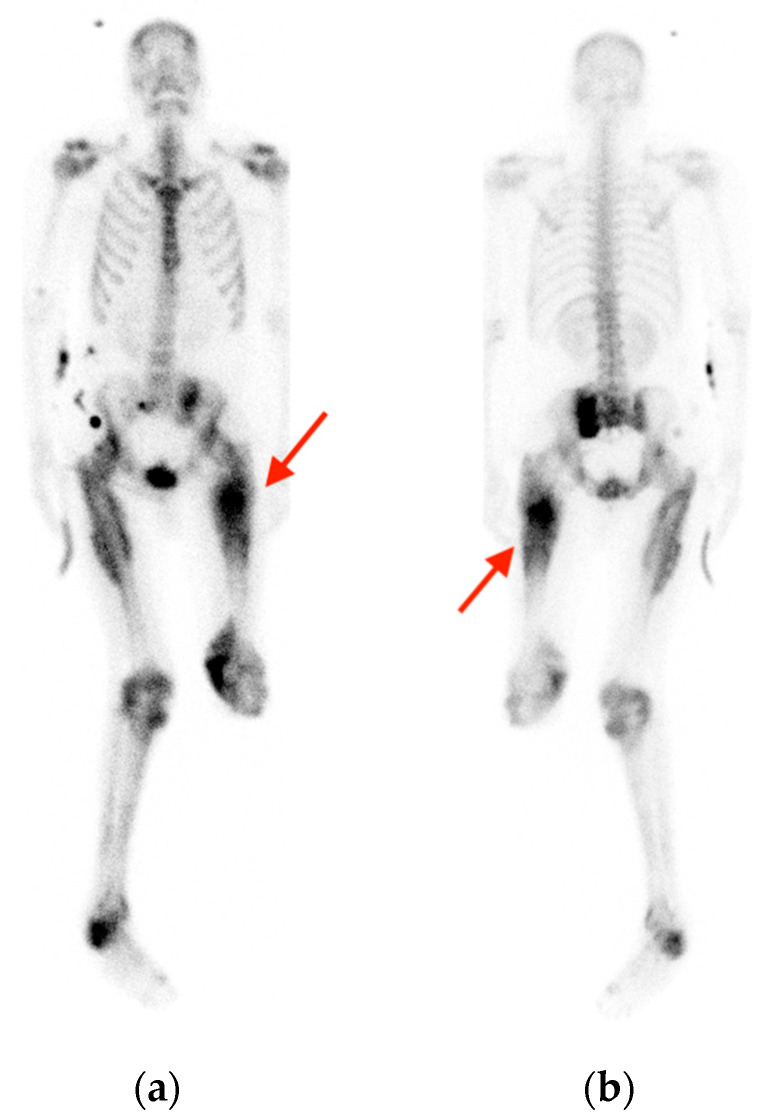
Anterior (**a**) and posterior (**b**) bone scintigraphy in a 41-year-old man with history of traumatic paraplegia and osteomyelitis shows asymmetric increased uptake in the soft tissues surrounding the left thigh (red arrows), later found to be consistent with an area of developing myositis ossificans.

Myositis ossificans (MO) is a dysregulated response most commonly related to muscular injury; however, in many cases a history of trauma need not be present [47]. Although the exact pathophysiology is not known, MO is understood to progress through three stages over the course of 12 or more weeks: early, intermediate, and mature [48]. Accordingly, radiologic findings will differ depending on the stage of MO (Figure 10). In theory, an area of MO will display uptake on bone scintigraphy until matured; however, MO lesions may display chronic uptake even after maturity [49]. FDG PET/CT will show corresponding increased uptake, with SUVs as high as 13, and a general decrease in uptake over time as the lesion matures [50]. MO should be distinguished by its zonal peripheral calcification that develops with increasing maturity of the lesion [51].

### 3.3. Calcific Tendinopathy

**Figure 11:**
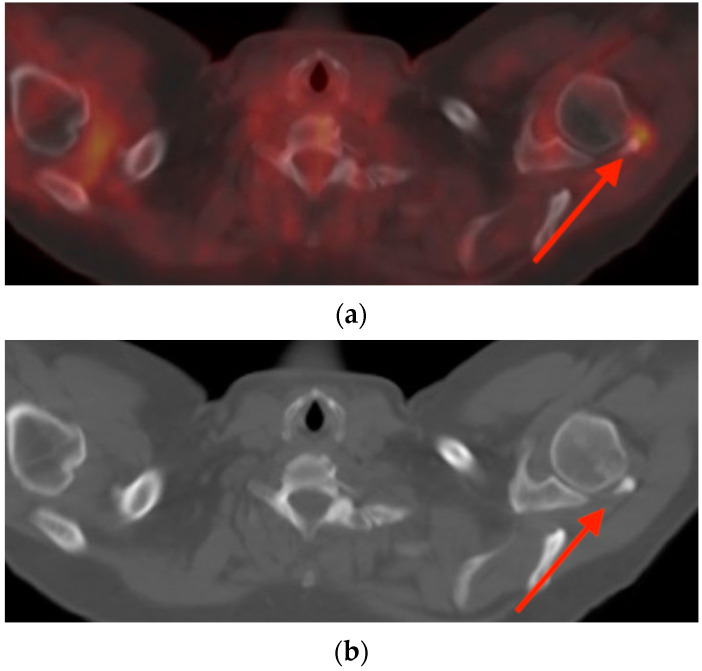
(**a**) Axial fused FDG PET/CT images in a 52-year-old woman with follicular lymphoma demonstrate a hypermetabolic focus adjacent to the posterolateral aspect of the left humeral head, with SUVmax 2.5 (red arrow). (**b**) Plain axial CT images better depict the amorphous calcification within the rotator cuff tendons (red arrow). The findings are consistent with calcific tendinopathy of the rotator cuff with associated inflammatory reaction within the surrounding tissues. CT = computed tomography. FDG PET/CT = fluorine-18 fluorodeoxyglucose positron emission tomography/computed tomography. SUVmax = maximum standard uptake value.

Calcific tendinopathy (CaT) involves the deposition of calcium phosphate crystals within tendons with a surrounding inflammatory reaction. Though it most commonly occurs in the rotator cuff tendons of the shoulder, particularly the supraspinatus [52], it has been found in a variety of tendons, including those of the gluteal muscles [53] as well as other lower extremity muscles that insert on the femur [54]. In up to 40% of cases, there may be osseous findings including cortical erosion, subchondral sclerosis, and bone marrow edema, and virtually all cases will display focal uptake on bone scintigraphy [54] and FDG PET/CT (Figure 11). The identification of amorphous calcium deposits within tendons at the enthesis will differentiate CaT from potentially malignant pathologies.

## 4. Joint

### 4.1. Tenosynovial Giant Cell Tumor/Pigmented Villonodular Synovitis

**Figure 12:**
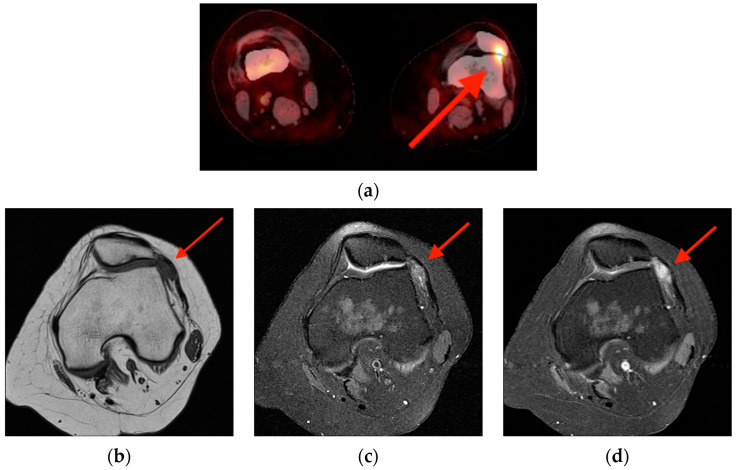
(**a**) FDG PET/CT obtained in a 43-year-old woman with head and neck cancer shows focal uptake within the lateral aspect of the left knee joint in the patellofemoral compartment (red arrow). This may be easily attributed to motion artifact or osteoarthritis; however, note the intra-articular location. MRI obtained eight months later shows an intra-articular soft tissue lesion with diffuse low signal intensity on T1-weighted images (**b**), heterogenous but primarily low signal intensity on T2-fat saturation imaged (**c**), and diffuse enhancement on post-contrast images (**d**), characteristic of TGCT/PVNS (red arrows). FDG PET/CT = fluorine-18 fluorodeoxyglucose positron emission tomography/computed tomography. MRI = magnetic resonance imaging. TCGT/PVNS = tenosynovial giant cell tumor/pigmented villonodular synovitis.

Tenosynovial giant cell tumor (TGCT) is a locally aggressive neoplasm within the synovium, bursa, and tendon sheath, historically referred to as pigmented villonodular synovitis (PVNS). TGCT/PVNS can have intense uptake on FDG PET/CT, with average maximum SUVs of 8.7 [55] to 11.3 [56]. The most characteristic feature of TGCT/PVNS is its intra-articular location, where it is exceedingly rare for a primary sarcoma or metastasis to originate. If focal intra-articular FDG uptake is associated with an isolated soft tissue mass on FDG PET/CT, MRI should be obtained for further evaluation, as TGCT/PVNS has a distinct heterogeneously low signal intensity on T1 and T2 weighted imaging due to hemosiderin deposits [57] and may occur more commonly than is generally appreciated [58] (Figure 12).

### 4.2. Adhesive Capsulitis

**Figure 13:**
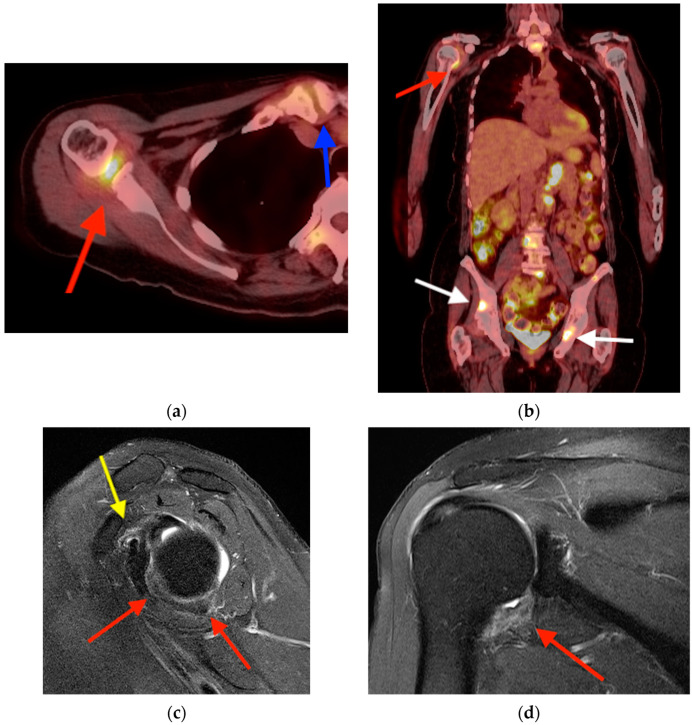
Axial (**a**) and coronal (**b**) FDG PET/CT images obtained in a 58-year-old woman with metastatic renal cell carcinoma show focal uptake in the right axillary recess (red arrows), along with additional areas of focal uptake at the site of known metastatic lesions (white arrows). There is osteoarthritis-related focal uptake at the right sternoclavicular joint (blue arrow). Axial (**c**) and coronal (**d**) T2-weighted MRI images show thickening of the joint capsule and inferior glenohumeral ligament at the axillary recess (red arrows) and effacement/obliteration of the coracoid fat and rotator interval with associated thickening of the glenohumeral ligament (yellow arrow), characteristic of adhesive capsulitis. FDG PET/CT = fluorine-18 fluorodeoxyglucose positron emission tomography/computed tomography. MRI = magnetic resonance imaging.

Adhesive capsulitis (AC) affects anywhere from 2 to 5% of the population and presents with acute or insidious shoulder pain [59]. Waldburger et al. found that almost 100% of bone scintigraphy studies will show uptake in the shoulder area; about one-third of uptake is diffuse, and about two-thirds of uptake is localized to the antero-medial border of the head of the humerus, the acromioclavicular joint, and the coracoid process [60] (Figure 13). Incidental AC has a prevalence of about 0.5% on FDG PET/CT, which will show C-shaped or dot-appearance focal uptake in the rotator interval, axillary recess, and anterior joint capsule, with SUVs up to 5.8 [61]. If PET/CT findings are equivocal, MRI can be performed to identify thickening and edema of the coracohumeral ligament, inferior glenohumeral ligament, and rotator interval joint capsule to confirm the diagnosis [62].

### 4.3. Acute Calcific Discitis

**Figure 14:**
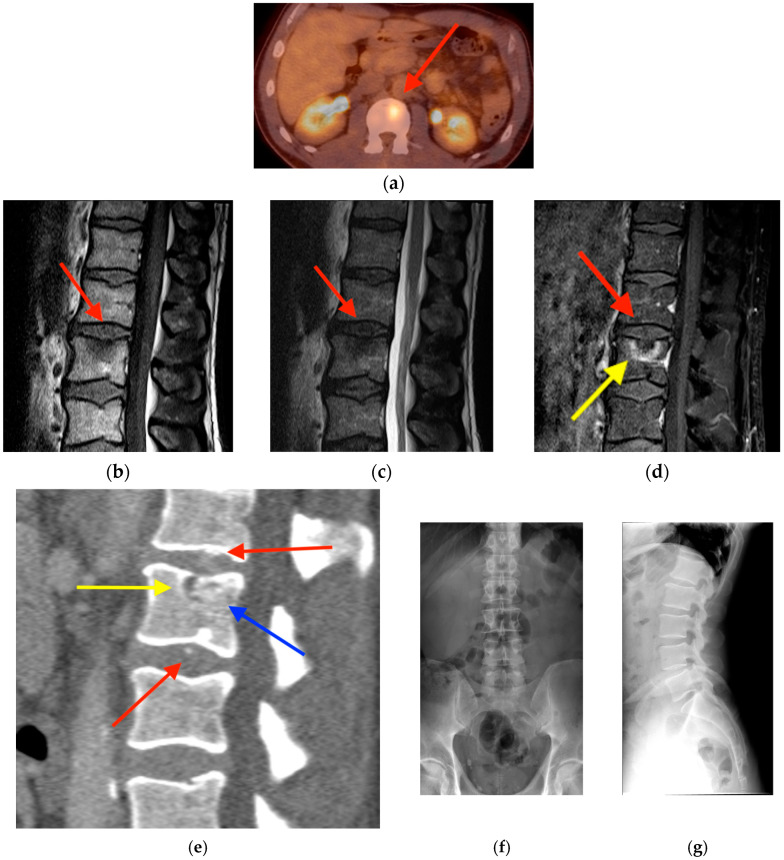
(**a**) FDG PET/CT obtained in a 61-year-old man with follicular lymphoma shows focal uptake within the L2 vertebral body (red arrow). MRI with T1-weighted (**b**), T2-fat saturation (**c**), and post-gadolinium T1-weighted (**d**) sequences shows faint hypointense intradiscal foci in all sequences (red arrows) and bone marrow enhancement (yellow arrow). (**e**) CT obtained one month later shows additional intradiscal calcifications (red arrows), one of which has migrated intraosseosly (blue arrow). There is focal cortical discontinuity along the superior endplate of L2 (yellow arrow). Frontal (**f**) and lateral (**g**) radiographs obtained thirty months later show complete reabsorption of the intradiscal calcifications with reconstitution of the superior endplate of L2 and preservation of disc spaces and vertebral body heights. Resolution on follow-up imaging distinguishes acute calcific discitis from Schmorl’s nodes, which do not resolve with time. CT = computed tomograpyhy. FDG PET/CT = fluorine-18 fluorodeoxyglucose positron emission tomography/computed tomography. MRI = magnetic resonance imaging.

Calcific discitis (CD) is an incredibly rare entity in adults, and most understanding of the disease is the result of studies in pediatric populations, in whom the disease is more common [63,64]. It is typically a self-limiting condition presenting with pain, fever, and elevated inflammatory markers that develops and resolves over the course of a few weeks, with a majority of cases resolved at 6 months [65]. Radiologically, it is characterized by disc calcifications, usually at one vertebral level, though it may affect multiple levels in 30–40% of cases [66]. This may be associated with endplate erosion and bone marrow edema on CT and MRI, respectively. CD can be differentiated from degenerative Schmorl’s nodes by the eventual reconstitution of vertebral endplates with no loss of body height. The appearance of CD on PET/CT has not been extensively studied; however, it is reasonable to surmise that FDG PET/CT would show focal uptake in the area of CD with gradual reduction in SUV as the CD resolves over weeks or months. One such example is illustrated in Figure 14.

## 5. Soft Tissue

### 5.1. Fat Necrosis

**Figure 15:**
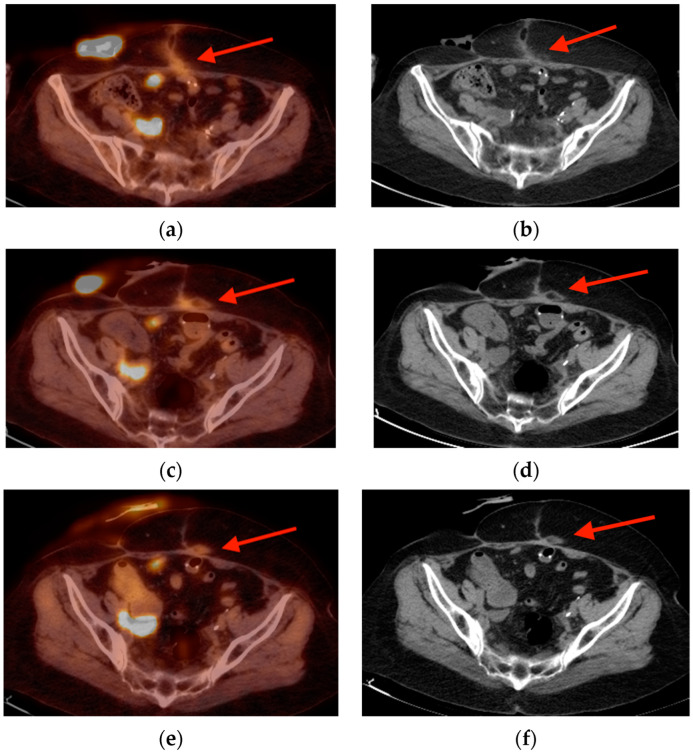
Multiple scans were obtained in a 72-year-old woman with bladder cancer after she had undergone surgical resection and reconstruction. Axial fused FDG PET/CT and non-fused CT images obtained at time 0 (**a**,**b**), 6 months later (**c**,**d**), and 12 months later (**e**,**f**) show evolution of a focal area of radiotracer uptake within the soft tissues of and anterior abdominal wall along the surgical scar, with macroscopic areas of fat (red arrows). CT = computed tomography. FDG PET/CT = fluorine-18 fluorodeoxyglucose positron emission tomography/computed tomography.

Fat necrosis is the result of disruption of the local blood supply of adipose cells and can result after trauma, surgery, biopsy, radiotherapy, and anticoagulation treatment. Its wide array of etiologies means that fat necrosis can be a common finding in patients with a vast number of cancers. Though commonly occurring in the breast, fat necrosis can be seen in almost any adipose tissue in the body including the soft tissues of the extremities. Imaging findings will vary with the age of the lesion, which may show variable SUV uptake ranging from 1.1 to 13.2 [67,68], though there is often identifiable macroscopic fat (Figure 15). Because it can occur along surgical scars or sites of trauma, fat necrosis may be mistaken for recurrent disease along a surgical bed.

### 5.2. Brown Fat Activation

**Figure 16:**
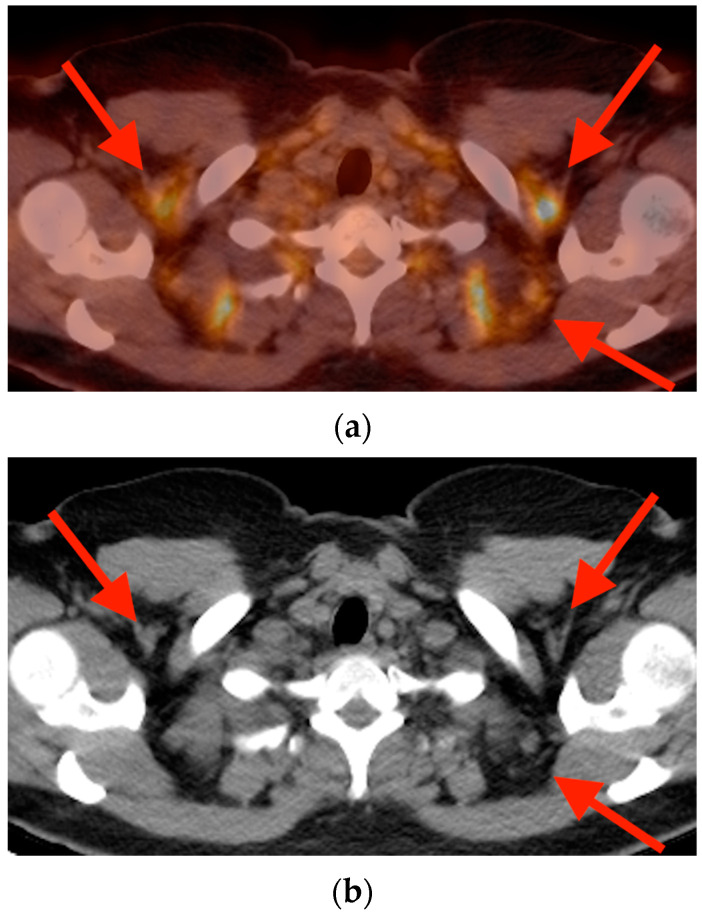
Axial FDG PET/CT fused (**a**) and non-fused CT (**b**) images obtained in a 41-year-old woman with cervical cancer demonstrate triangular-shaped areas of focal uptake in the cervical, supraclavicular, and paravertebral regions which correspond with low density areas of fat (red arrows), characteristic of brown fat activation. CT = computed tomography. FDG PET/CT = fluorine-18 fluorodeoxyglucose positron emission tomography/computed tomography.

In contrast to fat necrosis, brown fat activation is a physiologic process with a more diffuse distribution on FDG PET/CT. The SUV may be equally variable, ranging from 0.4 to 12.4 (mean 4.6) [69]; however, brown fat activation should be distinguished by its occurrence in characteristic locations in the cervical, supraclavicular, axillary, and paravertebral regions (Figure 16).

### 5.3. Hibernoma

**Figure 17:**
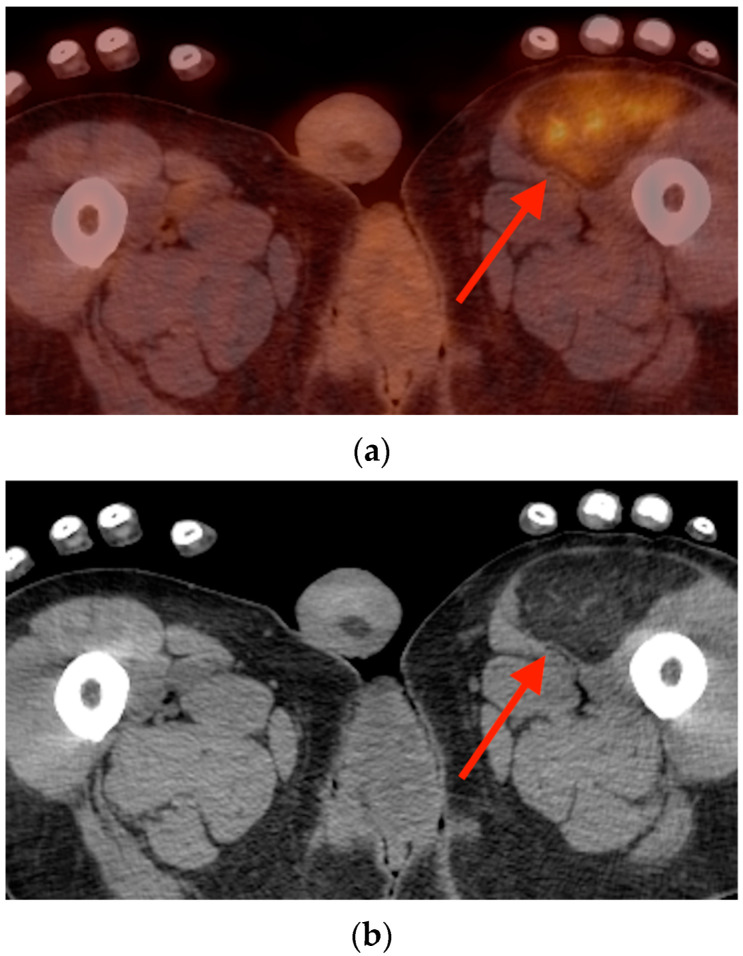
Axial fused FDG PET/CT (**a**) and plain CT (**b**) images obtained in a 54-year-old man with bladder cancer show an area of FDG uptake in the anterior left thigh, SUVmax 5.9, which corresponds with a well-circumscribed fat density lesion (red arrows). This was later biopsied, with pathology revealing a hibernoma. CT = computed tomography. FDG = fluorine-18 fluorodeoxyglucose. PET/CT = positron emission tomography/computed tomography.

Hibernomas and benign tumors of brown fat most commonly occur in the third decade of life [70]. Unlike physiologic brown fat activation, hibernomas will display focal, asymmetric, and mass-like FDG uptake within a well-circumscribed adipocytic tumor (Figure 17). Their SUVs have been reported to range from 2.7 to 24.3, sharing morphologic features of low-grade lipomatous tumors and functional metabolic activity with high-grade liposarcomas [71,72]. However, the combination of a purely lipomatous mass with strong FDG avidity (SUVmax > 10) is virtually pathognomonic for hibernoma [72]. Notably, hibernomas lack the *MDM2* amplification seen in well-differentiated liposarcoma/atypical lipomatous tumors [73].

## 6. Conclusions

There are a variety of benign incidental musculoskeletal conditions that can mimic neoplastic disease. As the number of oncology patients continues to grow, and the number of nuclear medicine scans along with them, there will be an increasing number of incidental pathologies found on these scans. Enhanced recognition of these disease entities may aid physicians in preventing unnecessary invasive procedures and providing reassurance to patients.

Similarly, imaging technology continues to advance and new radiopharmaceuticals, contrast agents, imaging protocols, and scanner types will continue to emerge. With regard to the findings described in this paper, imaging characteristics and clinical interpretation should not be impacted at different facilities provided image acquisition follows American College of Radiology (ACR) accredited facility guidelines, with appropriate radiopharmaceutical dose, camera peak, reconstruction, and post-processing. At the authors’ institutions, Omnipaque (iohexol) is used as an intravenous CT contrast agent and MultiHance (gadobenate dimeglumine) as an intravenous MRI contrast agent. With time, molecular imaging studies with receptor specific imaging such as iodine-131, fluoroestradiol F18, gallium 68/copper 64 DOTATATE, and gallium 68 prostate-specific membrane antigen (PSMA) may provide future clinical utility to clarify imaging findings and aid in diagnosis.
